# Acute exercise reduces feeding by activating IL-6/Tubby axis in the mouse hypothalamus

**DOI:** 10.3389/fphys.2022.956116

**Published:** 2022-11-14

**Authors:** Thayana de Oliveira Micheletti, Andressa Cassia dos Santos, Guilherme Zweig Rocha, Vagner Ramon Rodrigues Silva, Paula Gabriele Fernandes Quaresma, Heloisa Balan Assalin, Felipe Silva Junqueira, Eduardo Rochete Ropelle, Alexandre Gabarra Oliveira, Mario Jose Abdalla Saad, Patricia de Oliveira Prada

**Affiliations:** ^1^ School of Applied Sciences, State University of Campinas (UNICAMP), Limeira, Brazil; ^2^ Department of Internal Medicine, School of Medical Science, State University of Campinas (UNICAMP), Campinas, Brazil; ^3^ Department of Physical Education, Biosciences Institute, São Paulo State University (UNESP), Rio Claro, Brazil

**Keywords:** exercise, Tubby mouse, IL-6, JAK2, hypothalamus, food intake

## Abstract

**Background:** Acute exercise contributes to decreased feeding through leptin and interleukin/Janus kinase 2/signal transducers and activators of transcription 3 (IL-6/JAK2/STAT3) signaling. Considering the pleiotropic use of substrates by JAK2 and that JAK2 can phosphorylate the Tubby protein (TUB) in CHO-IR cells, we speculated that acute exercise can activate the IL-6/JAK2/TUB pathway to decrease food intake.

**Aims:** We investigated whether acute exercise induced tyrosine phosphorylation and the association of TUB and JAK2 in the hypothalamus and if IL-6 is involved in this response, whether acute exercise increases the IL-6/TUB axis to regulate feeding, and if leptin has an additive effect over this mechanism.

**Methods:** We applied a combination of genetic, pharmacological, and molecular approaches.

**Key findings:** The *in vivo* experiments showed that acute exercise increased the tyrosine phosphorylation and association of JAK2/TUB in the hypothalamus, which reduced feeding. This response was dependent on IL-6. Leptin had no additive effect on this mechanism.

**Significance:** The results of this study suggest a novel hypothalamic pathway by which IL-6 released by exercise regulates feeding and reinforces the beneficial effects of exercise.

## 1 Introduction

Due to the increasing global epidemic of obesity and its associated comorbidities such as type 2 diabetes (T2D) and cardiovascular diseases (González-Muniesa et al., 2017; Jiwani et al., 2019; Risk et al., 2020), more effective and lasting therapies should be implemented for these diseases. Being overweight and obese mainly result from increased consumption of high-calorie food and insufficient physical activity (Demmler et al., 2017; Bleich et al., 2018).

Exercise is key to the prevention of obesity and related diseases. Several studies demonstrated the beneficial effects of exercise in reducing adiposity, blood glucose, and blood pressure while increasing energy expenditure in individuals who are overweight or with T2D (Tuomilehto et al., 2001; Misra et al., 2008; Bray et al., 2016; Febbraio, 2017; Bleich et al., 2018; Myers et al., 2019). In addition to weight loss, physical exercise contributes to reducing appetite through leptin and interleukin 6 (IL-6) signaling in the hypothalamus (Ropelle et al., 2010; Timper et al., 2017; Smith, 2018; Pedersen, 2019).

Obesity leads to leptin resistance in the hypothalamus (Jais et al., 2017; Timper and Brüning, 2017). In this context, physical exercise can improve the leptin signaling pathway by activating Janus kinase 2/signal transducers and activators of transcription 3 (JAK2/STAT3), reducing food intake (Ropelle et al., 2010; Smith, 2018). IL-6 is released during physical exercise, leading to metabolic changes across tissues such as the liver, adipose tissue, and the hypothalamus (Ropelle et al., 2010; Ellingsgaard et al., 2011; Pedersen and Febbraio, 2012; Pedersen, 2019). The classical signal of IL-6 involves the membrane-bound alpha-receptor (IL-6R), leading to the dimerization of its co-beta-receptor glycoprotein 130 (gp130). Alternatively, IL-6 can also bind to soluble IL-6 receptors (sIL-6R) in cells that express gp130 but lack the IL-6R, allowing the stimulation of these cells in a process called trans-signaling. Both pathways activate JAK2 and STAT3 (Heinrich et al., 1998; Wallenius et al., 2002a; Wallenius et al., 2002b; Mauer et al., 2015; Bobbo et al., 2019). Thus, the effects of physical exercise on the action of leptin in the hypothalamus may depend on IL-6. However, considering the complexity and redundancy of the hypothalamic signaling pathway and the pleiotropic use of substrates by JAK2, IL-6 induced by physical exercise may also signal through JAK2 and other protein substrates to reduce food intake.

Nies et al. demonstrated a higher expression of the Tubby gene (*TUB*) in several regions of the central nervous system (CNS), including the hypothalamus, in human samples (Nies et al., 2018). Furthermore, mice with a *TUB* mutation develop obesity, insulin resistance, and a degeneration of the senses in adulthood (Coleman and Eicher, 1990; Kleyn et al., 1996; Stubdal et al., 2000). Due to *TUB* expression in tissues (Kleyn et al., 1996; Sahly et al., 1998; Nies et al., 2018) involved in the control of energy metabolism, we speculated that *TUB* might play a powerful role in regulating food intake (González-Muniesa et al., 2017; Lee et al., 2022). In the context of intracellular signaling, *in vitro* studies have shown that Tubby protein (TUB) may act as a transcription factor (Boggon et al., 1999) because it can translocate from the plasma membrane to the cellular nucleus (Santagata et al., 2001) upon hormone stimulation (Stretton et al., 2009). Furthermore, TUB has several tyrosine sites that were phosphorylated by JAK2 in CHO-IR cells (Kapeller et al., 1999). The results of these studies collectively suggested that, at least *in vitro*, TUB might act as a substrate of JAK2.

Based on the data above, we speculated in the present study that acute exercise can activate the IL-6/JAK2/TUB pathway to decrease food intake. Therefore, we investigated whether acute exercise induced tyrosine phosphorylation, the association of TUB and JAK2 in the hypothalamus, and the role of IL-6 in this response. We also evaluated whether the IL-6/TUB axis increased by acute exercise regulates feeding and if leptin had an additive effect on this mechanism.

## 2 Materials and methods

### 2.1 Animals and diet

The Ethics Committee of the State University of Campinas, which follows the Guide for the Care and Use of Laboratory Animals (published by the U.S. National Institute of Health no. 85-23 revised 1996), approved all animal protocols developed in the present study (CEUA 3704-1; 4626-1). All mice were maintained on 12 h/12 h light-dark cycles, starting at 06:00 a.m. (light cycle), and finishing at 06:00 p.m. when the dark cycle started. The room temperature was kept stable (22°C) with controlled humidity. The mice received a standard rodent chow (3.39 kcal/g; Nuvilab CR-1, Nuvital Quimtia, Brazil) and water *ad libitum*. We only used male mice. The University of São Paulo Central Breeding Center provided C3H/HeJ *Mus musculus* (IMSR Cat# JAX:000659, RRID: IMSR_JAX:000659). C3H/HePas laboratory mice (MGI Cat# 2160536, RRID:MGI:2160536) (Ropelle et al., 2010; Hua et al., 2018).

The University of Campinas Central Breeding Center provided the following mice: C57BL/6J *Mus musculus* (IMSR Cat# JAX:000664, RRID: IMSR_JAX:000664) and BKS.B6-Tubtub/Jng *Mus musculus* (IMSR Cat# JAX:004176, RRID:IMSR_JAX:004176) to make a colony. We applied the protocol described below to genotype the breeding colony of B6-*tub/tub* mice. Briefly, we purified genomic DNA from the tail tips by using a digestion buffer containing proteinase K. We used a Quant Studio 6 Flex Real-Time PCR System (#4485694, Applied Biosystems, CA, United States) to perform qPCR using the TaqMan technology. The reagents included Universal TaqMan Master Mix, TaqMan primers (10 µM), DNA sample (20 ng/µL), and Milli-Q H_2_O. The primer sequences were: F: 5′-TGT GAA GAA CTT CCA GAT CAT CCA-3′; R: 5′-GGC ACC ATG CGT ACA ACA TC-3′; 6FAM-GGC AAT GAC CTT GAG -MGBNFQ and VIC-GGC AAT GAC CGT GAG-MGBNFQ. The real-time PCR conditions consisted of 60°C for 30 s, 95°C for 10 min, then 35 cycles of 95°C for 15 s and 60°C for 1 min, and lastly, 60°C for 30 s. We obtained the following genotypes: wt/wt, B6-*tub/tub* (displaying a mutation in *TUB*), and the heterozygote (B6-*tub/+*). The present study used these three genotypes (Coleman and Eicher, 1990; Boggon et al., 1999; Wang et al., 2006).

All strains and ages are indicated in the experiments’ descriptions and Figure Legends.

### 2.2 Acute physical exercise

This protocol was performed as described previously (Silva et al., 2014; Lenhare et al., 2017; Silva et al., 2018). Briefly, the mice were acclimated to swimming for 2 days (10 min/24 h). The mice performed four 30-min exercise bouts, separated by a 5-min rest period with the water kept at 34–35°C. The mice swam in groups of four in a plastic barrel 40 cm in diameter filled to a depth of 20 cm. Acclimation and the exercise bouts of swimming were conducted during the light cycle and ended before the lights turned off (between 03:00 p.m. and 06:00 p.m.). The exercise protocol finished at 06:00 p.m., followed by the evaluation of the parameters described below.

### 2.3 Blood collection and ELISA measurements

Blood was collected after the exercise protocol ended after 06:00 p.m. (dark cycle). The mice were anesthetized (ketamine hydrochloride: 240 mg/kg and xylazine hydrochloride: 30 mg/kg *via* intraperitoneal) and euthanized by decapitation. The samples were centrifuged and serum was stored at −20°C until measurements. We determined IL-6 concentration by ELISA using a commercial kit (Crystal Chem Inc., Chicago, IL, United States) following the manufacturer’s instructions.

### 2.4 Surgery

The anesthetized mice were placed on the stereotaxic instrument (Ultra Precise—model 963—Kopf). We implanted one-side stainless steel guide cannulas (26-gauge, Plastics One) into the lateral ventricle of the hypothalamus at the following coordinates from the bregma: anterior/posterior: −0.5 mm, lateral: −1.3 mm, dorsoventral: −2.2 mm. After surgery, the mice were placed in individual cages. We measured their body weight and food intake for the following 7 days to evaluate their recovery from the surgery. We then confirmed the correct placement of the guide cannulas by intracerebroventricular (ICV) injection of 10 ng of angiotensin II. An immediate dipsogenic reaction within the first 10 min was expected for animals with the correct cannula placement. As described in our previous studies, we excluded mice in which angiotensin II injection did not elicit thirst (Caricilli et al., 2012; Weissmann et al., 2014; Quaresma et al., 2016; Quaresma et al., 2017; Zanotto et al., 2017; Campolim et al., 2020).

### 2.5 Intracerebroventricular injections

All injections were performed before the dark cycle started. The food intake measurements were performed during the dark cycle (after 06:00 p.m.), as indicated below in each protocol description. We injected a final volume of 1 µL of the solutions. The concentrations were previously tested elsewhere (Ropelle et al., 2010; Weissmann et al., 2014).

#### 2.5.1 ICV injection of recombinant IL-6

Male mice were injected with 100 ng of recombinant IL-6 (cat# I9646 Sigma-Aldrich, MDL number MFCD01656039) by ICV as described previously (Ropelle et al., 2010; Lenhare et al., 2017; Silva et al., 2018). ICV IL-6 or vehicle (Ropelle et al., 2010) was injected 30 min before food intake measurement or euthanasia, as shown in the timeline below.


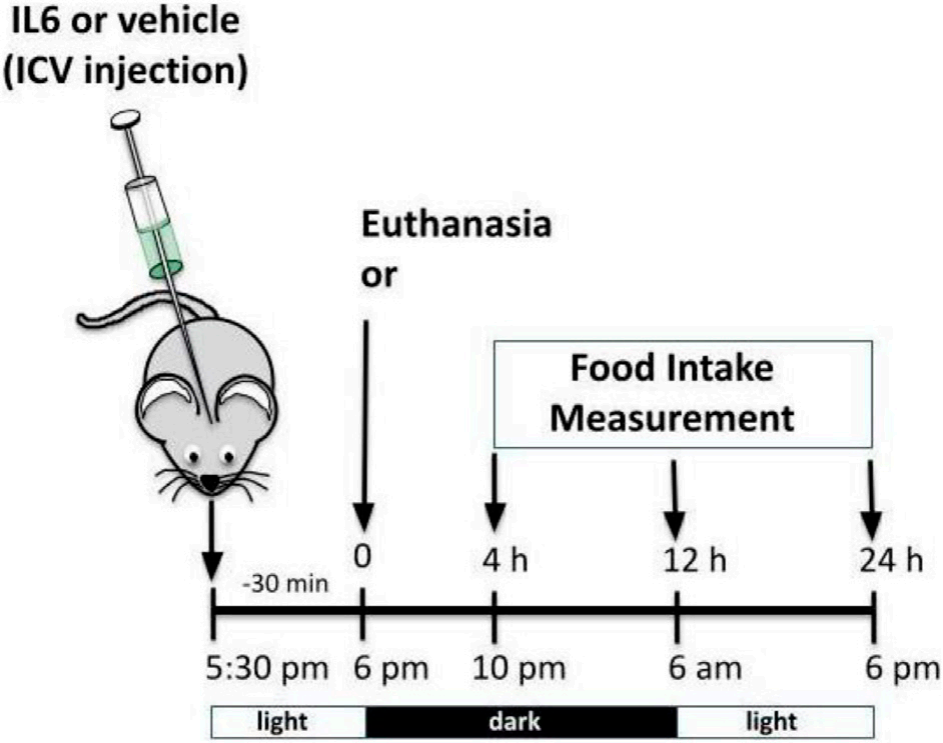


#### 2.5.2 ICV injection of AB IL-6

This protocol was based on those reported previously (Ropelle et al., 2010; Shirazi et al., 2013). To evaluate the role of IL-6 and TUB in feeding response after exercise, we used B6-*tub/tub* mice or the heterozygote B6-*tub/+* mice and injected a rabbit antiserum against IL-6 (ABIL-6) (25 ηg) (IL-6 (H-183) antibody (rabbit polyclonal; Santa Cruz Biotechnology Cat# sc-7920, RRID: AB_2127745) or its vehicle (rabbit pre-immune serum RPIS dissolved in saline) ICV 30 min before exercise and immediately after the exercise finished (light cycle). As previously described, we waited 15 min and started food intake measurements (dark cycle) (Ropelle et al., 2010).


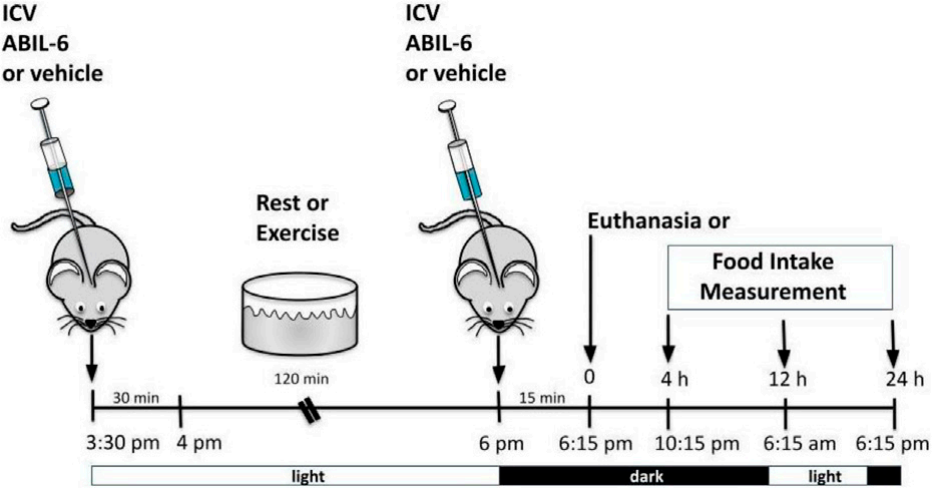


#### 2.5.3 ICV injection of AG490

ICV injection of AG490 (50 mM) (cat #0414, Tocris Bioscience, Bristol, United Kingdom) was used to inhibit JAK2 as previously described (Damm et al., 2013; Quaresma et al., 2017). ICV injection of AG490 or its vehicle was performed twice daily in the afternoon; 30 min later, we injected IL-6 ICV before the lights were turned off. Food intake measurements or euthanasia started after 06:30 p.m. (dark cycle). For this experiment, we used wt/wt mice (6–8 weeks of age) obtained from the B6-*tub/tub* mouse colony.


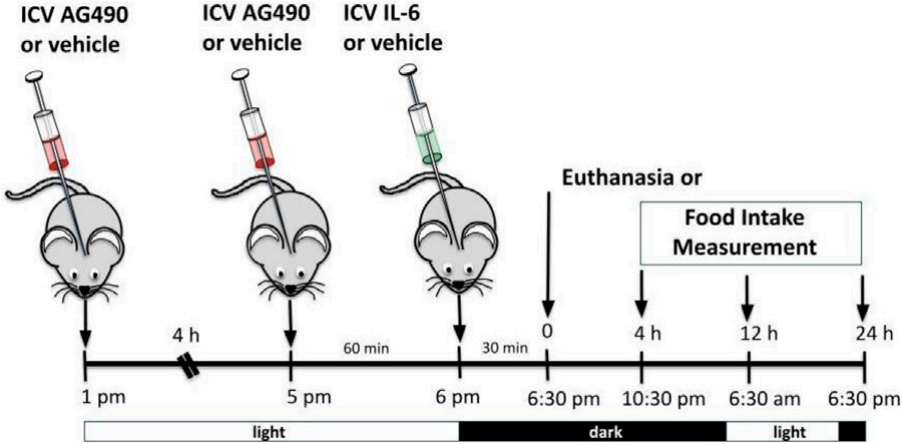


#### 2.5.4 ICV injection of leptin

ICV injection of recombinant leptin (10 ng/μL) (Calbiochem, San Diego, CA, United States) or saline was performed as prescribed previously (Caricilli et al., 2012; Weissmann et al., 2014; Quaresma et al., 2016; Quaresma et al., 2017; Campolim et al., 2020). IL-6 (ICV) or its vehicle was injected in a group of mice during the light cycle 30 min before leptin or saline injections. In another set of mice, the ICV injection of IL-6 was followed by acute exercise starting in the light cycle, followed by leptin injection. For all groups, food intake measurements or euthanasia occurred in the dark cycle, 15 min after leptin ICV injection.


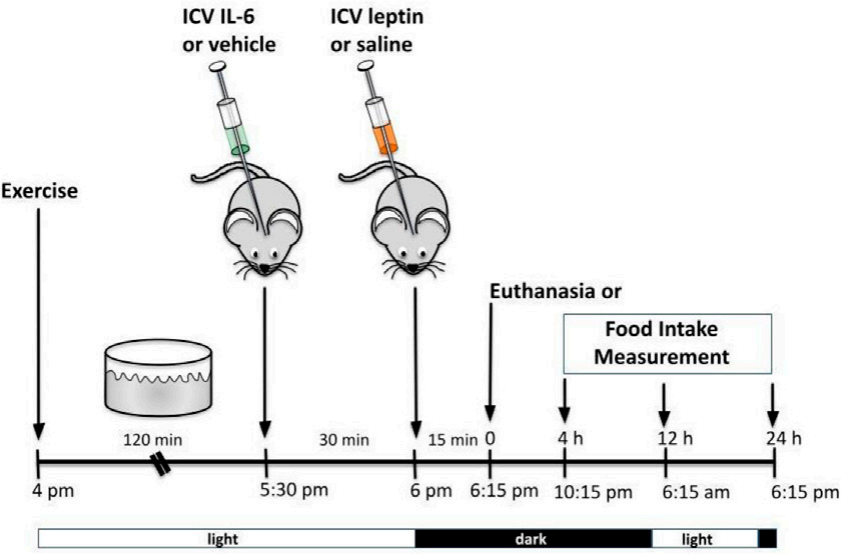


#### 2.5.5 ICV injection of ASO

We developed an antisense oligonucleotide (ASO) (5′AGG AAC ACC TTC TTG CCA T 3′) bought from IDT (Integrated DNA Technologies) to inhibit *TUB*. ASO or sense (control) was administered by ICV injections twice daily (08:30 a.m. and 05:00 p.m. during the light cycle) for four days. On the last day, sense and ASO were injected at 08:30 a.m. and 03:30 p.m. The mice were then injected with IL-6 or vehicle at 06:00 p.m. (before the lights turned off), 30 min before measuring food intake or euthanasia. For this experiment, we used wt/wt mice (6–8 weeks old) obtained from the B6-*tub/tub* mouse colony.


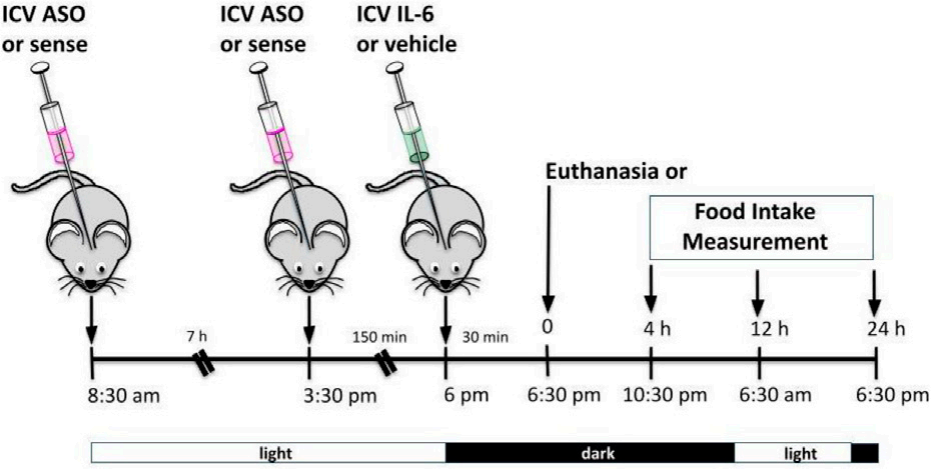


### 2.6 Immunoblotting analysis

**FIGURE 1:**
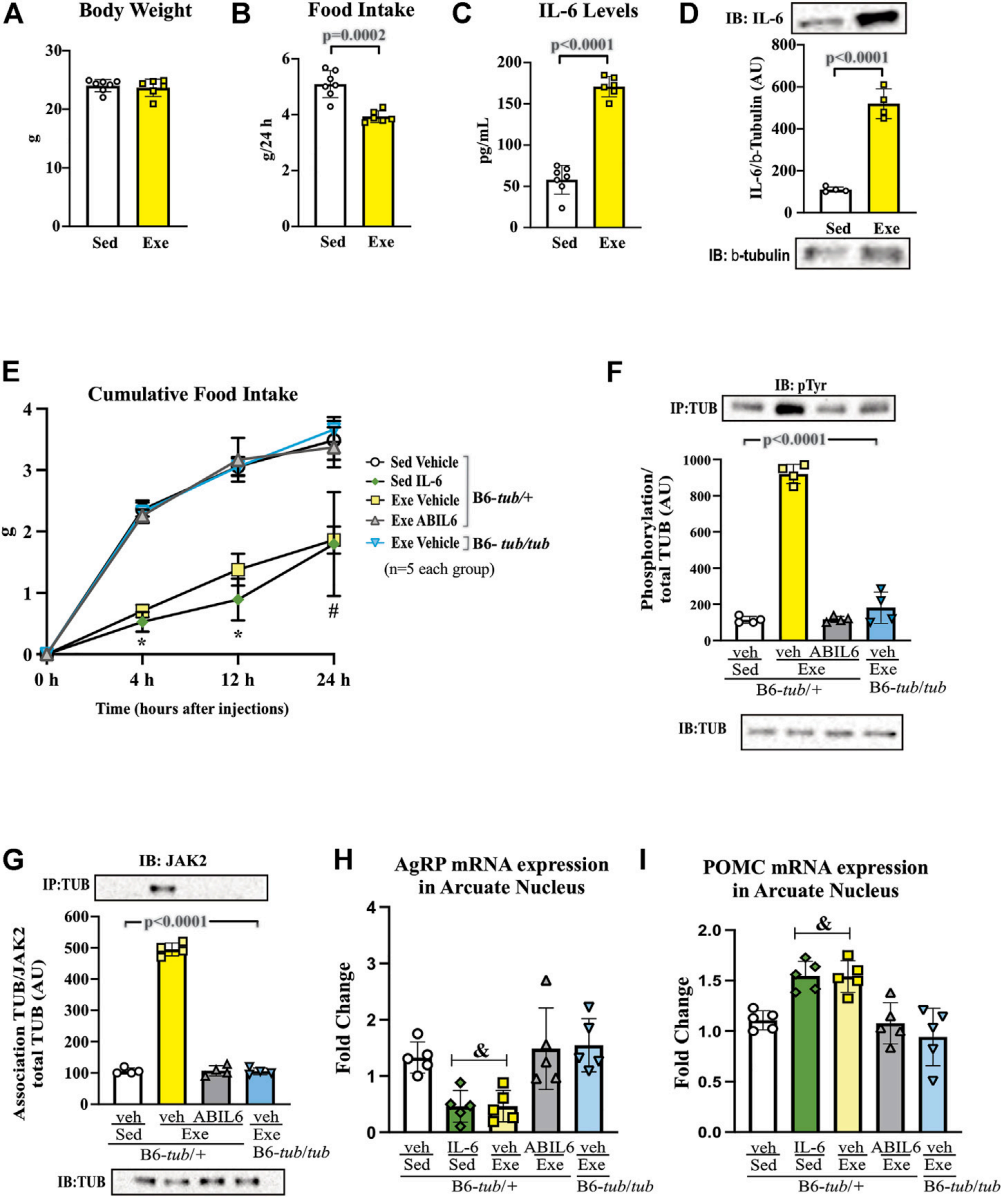
Effects of acute exercise on metabolic parameters and TUB regulation. **(A)** Body weight (g); **(B)** 24 h of food intake (g); **(C)** serum IL-6 levels (pg/ml); and **(D)** Western blot from hypothalamic lysates showing IL-6 expression in C57BL/6J mice (12–15 weeks of age) under resting conditions (Sed) or after swimming exercise (Exe). B6-*tub/+* or B6-*tub/tub* mice (6–8 weeks of age) were divided into the following groups: B6-*tub/+*: sedentary plus vehicle; sedentary plus IL-6 ICV; exercise plus vehicle; exercise plus ABIL6 (IL-6 antibody); B6-*tub/tub*: exercise plus vehicle. **(E)** Evaluation of the cumulative food intake (g) at 4 h, 12 h, and 24 h; **(H)** AgRP and **(I)** POMC mRNA expression in the arcuate nucleus of the hypothalamus. Western blot showing **(F)** TUB tyrosine phosphorylation and **(G)** TUB associated with JAK2 from hypothalamus lysates. The exercise was swimming. All mice were male. ICV: intracerebroventricular. All values are expressed as means ± standard deviation (SD). **(A–C)**: Sed (*n* = 7) and Exe (*n* = 6). **(D,F,G)**: (*n* = 4 each group). **(E,H,I)**: (*n* = 5 each group). Unpaired two-tailed *t*-tests were used to analyze **(A–D)**; two-way ANOVA was used to analyze **(E)**; one-way ANOVA with Tukey’s multiple comparisons tests was used to analyze **(F–I)**. *p < 0.0001 vs. other groups; &*p* < 0.05 vs. other groups; **#** [Sed + IL-6 vs. Sed + Veh (*p* = 0.0411); Sed + Veh vs. Exe + Veh (*p* = 0.0002); Sed + IL-6 vs. Exe (B6-*tub/tub*) (*p* = 0.0314); Exe + Veh vs. Exe (ABIL6) (*p* = 0.0004); Exe + Veh vs. Exe (B6-*tub/tub*) (*p* < 0.0001)].

**FIGURE 2:**
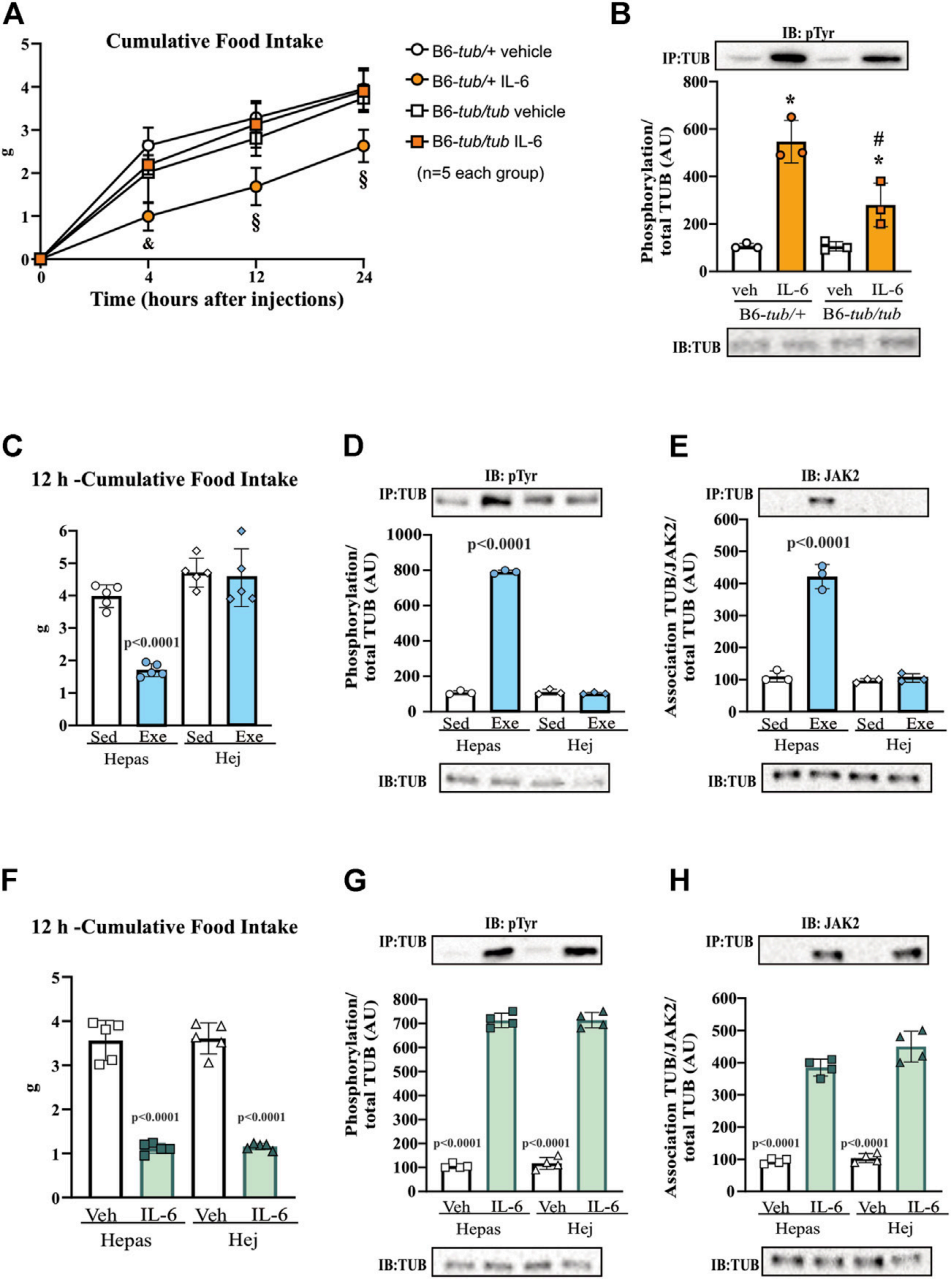
Effects of IL-6 on food intake and TUB regulation. **(A)** Evaluation of cumulative food intake (g) at 4 h, 12 h, and 24 h (*n* = 5 each group); Western blot showing **(B)** TUB tyrosine phosphorylation from hypothalamus lysates from young (6–8 weeks of age) heterozygous (B6-*tub/+*) or homozygous (B6-*tub/tub*) Tubby mice with vehicle or IL-6 ICV injection. **(C)** 12 h of cumulative food intake (g) (*n* = 5 each group); Western blot showing **(D)** TUB tyrosine phosphorylation, and **(E)** TUB associated with JAK2 from hypothalamus lysates from lean C3H/Hepas or C3H/HeJ mice under resting conditions (Sed) or after swimming exercise (Exe). **(F)** 12 h of cumulative food intake (g) (*n* = 5 each group); Western blot showing **(G)** TUB tyrosine phosphorylation, and **(H)** TUB associated with JAK2 from hypothalamus lysates from lean C3H/Hepas or C3H/HeJ mice treated with vehicle or IL-6 ICV injection. ICV: intracerebroventricular. We used only male mice. All values are expressed as means ± standard deviation (SD). Western blot assays (*n* = 3–5 in each group). Two-way ANOVA with Tukey’s multiple comparisons test was used for analyzing all data. **(A) &**B6-*tub/+*plus Veh vs. B6-*tub/+*plus IL-6 (*p* = 0.0007); B6-*tub/+*plus IL-6 vs. B6-*tub/tub* plus IL-6 (*p* = 0.0012); **§** B6-*tub/+*plus Veh vs. B6-*tub/+*plus IL-6 (*p* < 0.0068); B6-*tub/+*plus IL-6 vs. B6-*tub/tub* plus Veh or IL-6 (*p* < 0.0125). **(B)** *IL-6 increases TUB phosphorylation independently of genotypes (*p* = 0.0006); **#** B6-*tub/+*plus IL-6 vs. B6-*tub/tub* plus IL-6 (*p* = 0.0370). **(C)**, exercise in the Hepas group decreases food intake compared to other groups (*p* < 0.0001). **(D,E)** exercise in the Hepas group increases TUB phosphorylation and association with JAK2 compared to other groups (*p* < 0.0001). **(F)** IL-6 decreases food intake independently of the genotype compared to sedentary groups (*p* < 0.0001). **(G,H)** IL-6 in the Hepas and Hej groups increases TUB phosphorylation and association with JAK2 compared to the sedentary groups (*p* < 0.0001).

**FIGURE 3:**
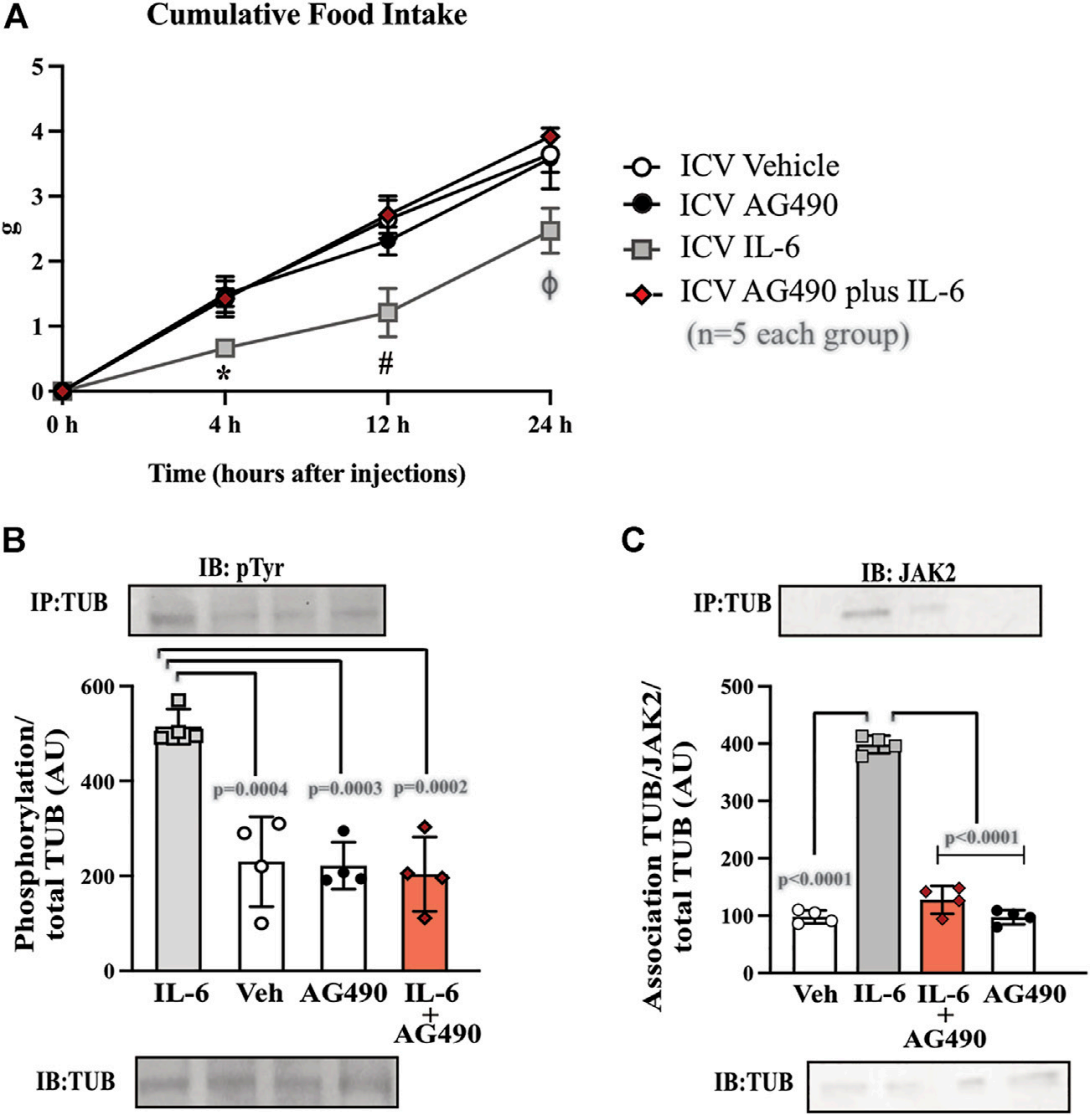
Hypothalamic IL-6 action through JAK2 to activate TUB. **(A)** Evaluation of cumulative food intake (g) at 4 h, 12 h, and 24 h (*n* = 5 in each group); Western blot showing **(B)** TUB tyrosine phosphorylation and **(C)** TUB associated with JAK2 from hypothalamic lysates from wt/wt mice (6–8 weeks old) obtained in the colony of B6-*tub/tub* mice under resting conditions (Sed). The mice were treated with vehicle or AG490 (JAK2inhibitor), IL-6, or IL-6 with AG490 by intracerebroventricular (ICV) injections. We used male mice. All values are expressed as means ± standard deviation (SD). Western blot assays (*n* = 4 in each group). Two-way ANOVA was used to analyze A, while one-way ANOVA with Tukey’s multiple comparisons tests was used to analyze B and C. *ICV IL-6 vs. Veh, AG490, and IL-6 plus AG490 (*p* < 0.0078); **#**ICV IL-6 vs. Veh, AG490 and IL-6 plus AG490 (*p* < 0.0041); ϕ ICV IL-6 vs. Veh, AG490, and IL-6 plus AG490 (*p* < 0.0136).

**FIGURE 4:**
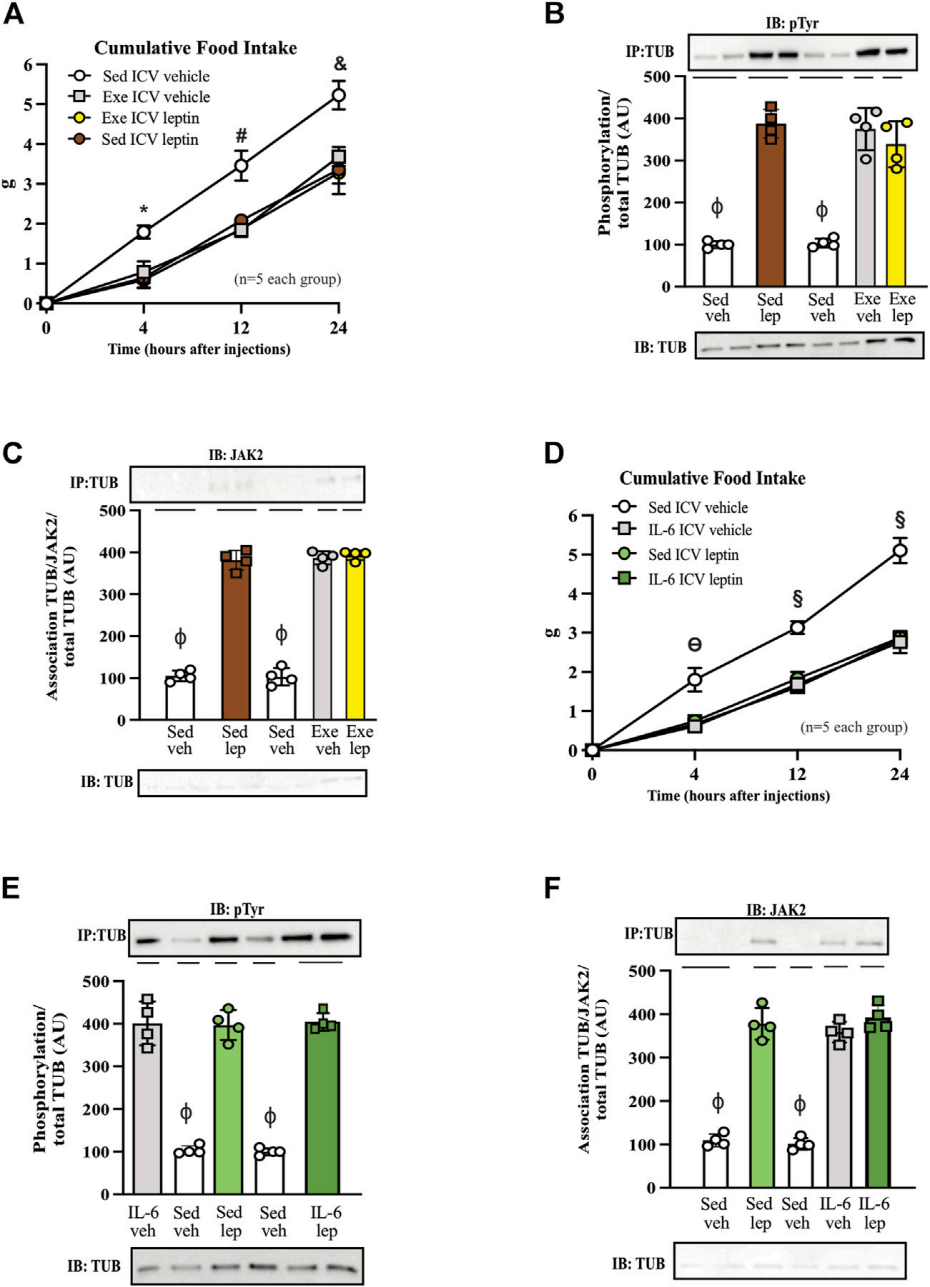
Leptin has no additive effect on food intake and TUB signaling in exercised mice or mice treated with IL-6. **(A)** Evaluation of cumulative food intake (g) at 4 h, 12 h, and 24 h; Western blot showing **(B)** TUB tyrosine phosphorylation and **(C)** TUB associated with JAK2 from hypothalamus lysates from C57BL/6J mice (11–12 weeks of age) under resting conditions (Sed) or after swimming exercise (Exe) treated with vehicle or leptin *via* intracerebroventricular (ICV). In another independent experiment, we also used C57BL/6J mice (11–12 weeks of age) only under resting conditions (Sed) treated with vehicle or IL-6 and leptin *via* intracerebroventricular (ICV). We analyzed **(D)** cumulative food intake (g) at 4 h, 12 h, and 24 h; Western blot showing **(E)** TUB tyrosine phosphorylation and **(F)** TUB associated with JAK2 from hypothalamus lysates. We used male mice. All values are expressed as means ± standard deviation (SD). Western blot assays (*n* = 4 in each group). Two-way ANOVA was used to analyze **(A,D)**, while one-way ANOVA with Tukey’s multiple comparisons tests was used to analyze **(B,C).** * Sed Veh vs. Exe Veh, Sed leptin, and Exe leptin (*p* < 0.0011); **#** Sed Veh vs. Exe Veh, Sed leptin, and Exe leptin (*p* < 0.0024); **&** Sed Veh vs. Exe Veh, Sed leptin, and Exe leptin (*p* < 0.0012); **ϴ** Sed Veh vs. IL-6 Veh, Sed leptin, and IL-6 leptin (*p* < 0.0017); **§** Sed Veh vs. IL-6 Veh, Sed leptin, and IL-6 leptin (*p* < 0.0001); ϕp<0.0001 vs. other groups.

We anesthetized mice using a mixture of ketamine hydrochloride (240 mg/kg) and xylazine hydrochloride (30 mg/kg) administered intraperitoneally. After the mice lost their reflexes, we euthanized them by decapitation. Their hypothalami were quickly dissected, placed into microfuge tubes, snap-frozen by immersing the tubes in liquid nitrogen (N_2_), and stored at −80°C until future processing. For processing, the samples were homogenized in freshly prepared ice-cold buffer (100 mM Tris-HCl; 10 mM EDTA; 10 mM sodium pyrophosphate; 100 mM NaF; 10 mM sodium orthovanadate; 2 mM phenylmethylsulfonyl fluoride; 1% Triton X-100; 0.1 mg/ml aprotinin [Sigma-Aldrich]) (Weissmann et al., 2014). Immunoprecipitation (IP) and immunoblotting (IB) were performed as previously described (Prada et al., 2005; Caricilli et al., 2012; Weissmann et al., 2014; Quaresma et al., 2016; Quaresma et al., 2017; Zanotto et al., 2017; Campolim et al., 2020) and used the BCA assay to determine the protein concentrations. The total protein concentrations were then normalized between the lysates (1000 μg of protein/lysate) for IP. We used a Tub (M-19) antibody against the C-terminal (goat polyclonal; Santa Cruz Biotechnology cat# sc-1959, RRID: AB_671393). A similar antibody from Santa Cruz against C-terminal was previously validated and published (Koritschoner et al., 2001; Stretton et al., 2009; Kim et al., 2017). The following antibodies were also used and previously validated and published (Prada et al., 2005; Ropelle et al., 2010; Weissmann et al., 2014): p-Tyr (PY20) antibody (mouse monoclonal; Santa Cruz Biotechnology cat# sc-508, RRID:AB_628122), JAK2 (HR-758) antibody (rabbit polyclonal; Santa Cruz Biotechnology cat# sc-278, RRID: AB_631853), IL-6 (H-183) antibody (rabbit polyclonal; Santa Cruz Biotechnology cat# sc-7920, RRID: AB_2127745), and beta-tubulin antibody (rabbit polyclonal; Cell Signaling Technology cat# 2146, RRID: AB_2210545). We used UN-SCAN-IT gel software to determine the band intensities. The raw immunoblotting data for Figures 1–4 are attached at the end of the manuscript.

### 2.7 Gene expression

Twelve hours post-exercise, the mice were euthanized for dissection of the arcuate nucleus of the hypothalamus, as described elsewhere (Katashima et al., 2022). We performed a quick harvest, placed the samples in N_2_, and stored them at −80°C until RNA analysis. We used a QIAcube instrument (cat# 9002864; Qiagen, Inc., CA, United States) for fully automated nucleic acid extraction with Qiagen’s spin-column kits (RNeasy Mini kit, cat#7 4106; Qiagen, Inc., CA, United States). A Nanodrop 2000 instrument was used to quantify the RNA yield. All samples with 2 µg of total RNA were used to produce cDNA (cat# 4368814, High-capacity cDNA Reverse Transcription Kit, Applied Biosystem, CA, United States). A QuantStudio 6 Flex real-time PCR system (cat #4485694, Applied Biosystems, CA, United States) was used to perform the qPCR on duplicate samples using Universal TaqMan® Master Mix, TaqMan® primers (10 µM), cDNA sample (100 ηg), and Milli-Q H_2_O. The primer sequences were obtained from Life Technologies (CA, United States): Pomc, Mm00435874_m1 and Agrp, Mm00475829_g1. DataAssist™ software (Applied Biosystems, CA, United States) was used to determine the cycle threshold for each sample and the relative expression levels following normalization to GAPDH (Mm00475829_g1), hypoxanthine-guanine phosphoribosyltransferase (Hprt, Mm01545399_m1), and beta-actin (Actb, Mm00607939_m1).

We used fold_change to express mRNA levels, and calculated data using the ddCt method.

### 2.8 Food intake measurements

The mice were housed singly in their home cages with bedding. We weighed their food before and after the experiment, following the timelines described above. We also weighed any food left in the cages (Caricilli et al., 2012; Weissmann et al., 2014; Quaresma et al., 2016; Quaresma et al., 2017; Campolim et al., 2020).

### 2.9 Statistical analysis

All values are expressed as means ± standard deviation (SD). We used GraphPad Prism (San Diego, CA, United States) to analyze the data and prepare the graphics. As appropriate, two-tailed Student t-test or one- and two-way ANOVA with Tukey post-tests were employed as statistical tests. Differences were considered significant for *p* < 0.05. We referred to the statistical analysis and the N in the figure legends.

## 3 Results

### 3.1 Acute physical exercise induces tyrosine phosphorylation of TUB and its association with JAK2 in the hypothalamus *via* IL-6 *in vivo* to reduce food intake

A single session of exercise in lean mice (C57BL/6 12–15 weeks of age) showed no changes in body weight (Figure 1A).

This result is consistent with previous data from our group, in which acute exercise did not change the body weight of rodents (Ropelle et al., 2010). After the end of the exercise in the present study, we observed a reduction in food intake that lasted up to 24 h (Figure 1B), reinforcing the anorexigenic effect of the acute exercise (Ropelle et al., 2010). We also observed elevated serum levels (Figure 1C) and increased protein expression of IL-6 in the hypothalamus of the mice (Figure 1D and Supplementary Figure S7).

Sedentary control mice treated with IL-6 ICV were previously reported to show reduced food intake compared to sedentary controls that received vehicle injections *via* ICV (Timper et al., 2017; Silva et al., 2018). To investigate whether Tub participates in the anorexigenic effect of acute exercise due to IL-6, we treated control mice (B6- *tub/+*) with an anti-IL-6 antibody or vehicle before starting the exercise protocol and used a B6- *tub/tub* (displaying a mutation in *TUB*) after exercise. Therefore, we analyzed the following groups: B6- *tub/+* sedentary + vehicle; B6- *tub/+* sedentary + IL-6; B6- *tub/+* exercise + vehicle; B6- *tub/+* exercise + anti-IL-6 antibody; and B6- *tub/tub* mice exercise + vehicle. We applied two-way ANOVA to evaluate the cumulative food intake at 4, 12, and 24 h after the single exercise session. As expected, the animals who performed exercise animals and were administered vehicle or sedentary mice treated with IL-6 showed reduced food intake compared to non-exercised animals (sedentary group). This effect was inhibited by pre-treatment with an anti-IL-6 antibody (Figure 1E). To investigate whether TUB regulated food intake following exercise, we evaluated young, lean (22.2 ± 0.4 g), and normoglycemic (82 ± 10 mg/dl) B6- *tub/tub* mice after exercise. In mice with a *TUB* mutation, exercise significantly increased serum levels of IL-6 (ρg/mL) (sedentary: 25.33 ± 3.08; *n* = 4 and exercised: 49.47 ± 8.25; *n* = 4; unpaired *t*-test *p* = 0.0336) but did not reduce food intake, as observed in the exercised control mice (Figure 1E).

Regarding intracellular signaling, acute physical exercise increased tyrosine phosphorylation of TUB in the hypothalamus of control mice, which was prevented by pre-treatment with an anti-IL-6 antibody (Figure 1F and Supplementary Figure S7). Likewise, B6-*tub/tub* mice that had performed exercise showed mitigated tyrosine phosphorylation of TUB in the hypothalamus compared to control mice that had performed acute exercise (Figure 1F and Supplementary Figure S7). The association of TUB/JAK2 increased with physical exercise in the control mice. However, pre-treatment with an anti-IL-6 antibody and a *TUB* mutation suppressed the effect of acute exercise on increasing the TUB/JAK2 association (Figure 1G and Supplementary Figure S7). These results showed that acute physical exercise induced tyrosine phosphorylation of TUB and its association with JAK2 in the hypothalamus and suggested that IL-6 is involved in this response *in vivo*. Therefore, our data demonstrated that the hypothalamic IL-6/TUB axis might play a role in controlling food intake *via* acute physical exercise.

Furthermore, 12 h after the physical activity, we evaluated AgRP and POMC mRNA levels in the arcuate nucleus of the hypothalamus as these neuropeptides are directly involved in food intake regulation after physical exercise (Bunner et al., 2020). We observed decreased AgRP mRNA levels compared to those in the sedentary group, the exercised group pre-treated with ABIL6, and B6-*tub/tub* mice (Figure 1H). In contrast, POMC mRNA levels were increased compared to those in the sedentary group (Figure 1H).

### 3.2 Hypothalamic axis IL-6/TUB is essential for the regulation of food intake

To confirm the role of TUB in hypothalamic IL-6 signaling, we treated B6-*tub/tub* mice or heterozygous control mice (B6-*tub/+*) with IL-6 or vehicle and measured their food intake for 4, 12, and 24 h. The results of the two-way ANOVA showed that, as expected, the ICV injection with IL-6 decreased food intake in control mice compared to vehicle treatment. In contrast, the *TUB* mutation impaired the anorexigenic effect of IL-6 because B6-*tub/tub* mice did not show decreased food ingestion after IL-6 injection (Figure 2A). These data suggested that IL-6 signaling required TUB to exert its anorexigenic effect. In the molecular context, IL-6 increased tyrosine phosphorylation of TUB in the hypothalamus of control mice compared to vehicle ICV injection. Although treatment with IL-6 by ICV injection in B6-*tub/tub* mice significantly increased the tyrosine phosphorylation of TUB, this effect was lower than the effect of IL-6 in the control mice (Figure 2B and Supplementary Figure S7).

The results of two-way ANOVA showed that ASO treatment impaired the anorexigenic effect of IL-6 compared to mice treated with sense or with ASO without IL-6 (Figure 6 and Supplementary Material). Moreover, IL-6 increased tyrosine phosphorylation of TUB and the association with JAK2 in the hypothalamus of mice treated with sense, while treatment with ASO blunted these responses (Figures 6B,C and Supplementary Material). Collectively, these data reinforced the fundamental role of the IL-6/TUB axis in controlling food intake.

We used C3H/HeJ mice with a deficient IL-6 response to exercise (Ropelle et al., 2010; Hua et al., 2018) to investigate the relationship between exercise, IL-6, and TUB pathways in the hypothalamus, with C3H/Hepas as control mice. C3H/HeJ mice have reduced hypothalamic IL-6 levels after exercise (Ropelle et al., 2010). Two-way ANOVA showed that acute exercise decreased 12 h-food intake in C3H/Hepas mice compared to the sedentary group of C3H/Hepas mice. In contrast, exercised C3H/HeJ mice did not show decreased food intake, similar to the sedentary groups (Figure 2C). In C3H/Hepas mice, acute exercise increased TUB tyrosine phosphorylation in the hypothalamus compared to other groups (Figure 2D and Supplementary Figure S7). In C3H/HeJ mice, exercise did not increase TUB tyrosine phosphorylation. Similar results were observed in the association of TUB/JAK2 in the hypothalamus. The exercise improved the association of TUB/JAK2 in the hypothalamus of C3H/Hepas mice compared to other groups. However, C3H/HeJ mice did not show an increased association of TUB/JAK2 in response to exercise, instead showing the same pattern observed in the sedentary groups (Figure 2E and Supplementary Figure S7). In another set of experiments using C3H/HeJ and C3H/Hepas mice, instead of exercise, we injected IL-6 or vehicle *via* ICV and followed their feeding for 12 h. Two-way ANOVA demonstrated that IL-6 ICV injection decreased food intake in both C3H/Hepas and C3H/HeJ mice compared to vehicle-treated mice (Figure 2F). Accordingly, TUB tyrosine phosphorylation and TUB/JAK2 association increased in the hypothalamus in response to IL-6 ICV injection in both C3H/Hepas and C3H/HeJ mice compared to vehicle-treated mice (Figures 2G,H and Supplementary Figure S7).

### 3.3 Hypothalamic IL-6 acts through JAK2 to induce TUB tyrosine phosphorylation

To investigate the link between the anorexigenic effect of IL-6 signaling and the interaction of TUB/JAK2, we inhibited JAK2 with ICV AG490 before treatment with IL-6 and followed food intake for 4, 12, and 24 h. The ICV injection of AG490 alone did not alter food intake. However, AG490 injected before IL-6 prevented the anorexigenic effect of IL-6 (Figure 3A, two-way ANOVA). In the context of intracellular signaling, IL-6 ICV injection increased tyrosine phosphorylation of TUB in the hypothalamus, which was prevented by pretreatment with AG490 (Figure 3B and Supplementary Figure S7). Likewise, the association of TUB with JAK2 was increased by IL-6 but was prevented by pretreatment with AG490 (Figure 3C and Supplementary Figure S7).

### 3.4 Leptin has no additive effect on food intake and TUB signaling in exercised mice or mice treated with IL-6

Figure 4A shows that both leptin and exercise resulted in decreased food intake and that adding leptin to exercise had no additive effect on food intake after 4, 12, and 24 h. The ICV injection of leptin increased the tyrosine phosphorylation of TUB in the hypothalamus independently of animal activity (sedentary or exercised). Exercise with saline and leptin showed similar increases in the tyrosine phosphorylation of TUB compared to those in the sedentary saline groups (Figure 4B and Supplementary Figure S8).

Similarly, leptin increased the TUB/JAK2 association in the hypothalamus independent of mouse activity. Exercise with saline and leptin similarly increased the TUB/JAK2 association compared to those in the sedentary saline groups (Figure 4C and Supplementary Figure S8). Both results (Figures 4B,C) suggested that leptin had no additive effect on exercising.

To investigate whether IL-6 might show similar effects as in the exercised mice in response to leptin, we treated sedentary animals with IL-6 or saline. We observed that IL-6 or leptin alone or IL-6 plus leptin induced an anorexigenic effect starting at 4 hours and persisting for up to 24 h (Figure 4D).

In the molecular context, the ICV injection of IL-6 plus leptin increased the tyrosine phosphorylation of TUB in the hypothalamus of sedentary mice injected with saline as much as IL-6 or leptin alone. We observed no differences among IL-6 alone, leptin alone, or IL-6 plus leptin injections on TUB tyrosine phosphorylation (Figure 4E and Supplementary Figure S8). Similarly, leptin increased the TUB/JAK2 association compared to saline. In mice injected with IL-6, saline or leptin injections increased the TUB/JAK2 association. We did not observe differences among IL-6 alone, leptin alone, or IL-6 plus leptin injections on TUB/JAK2 association (Figure 4F and Supplementary Figure S8).

### 3.5 Knock-down of TUB expression in the hypothalamus mitigates the effect of IL-6 on food intake

**FIGURE 5:**
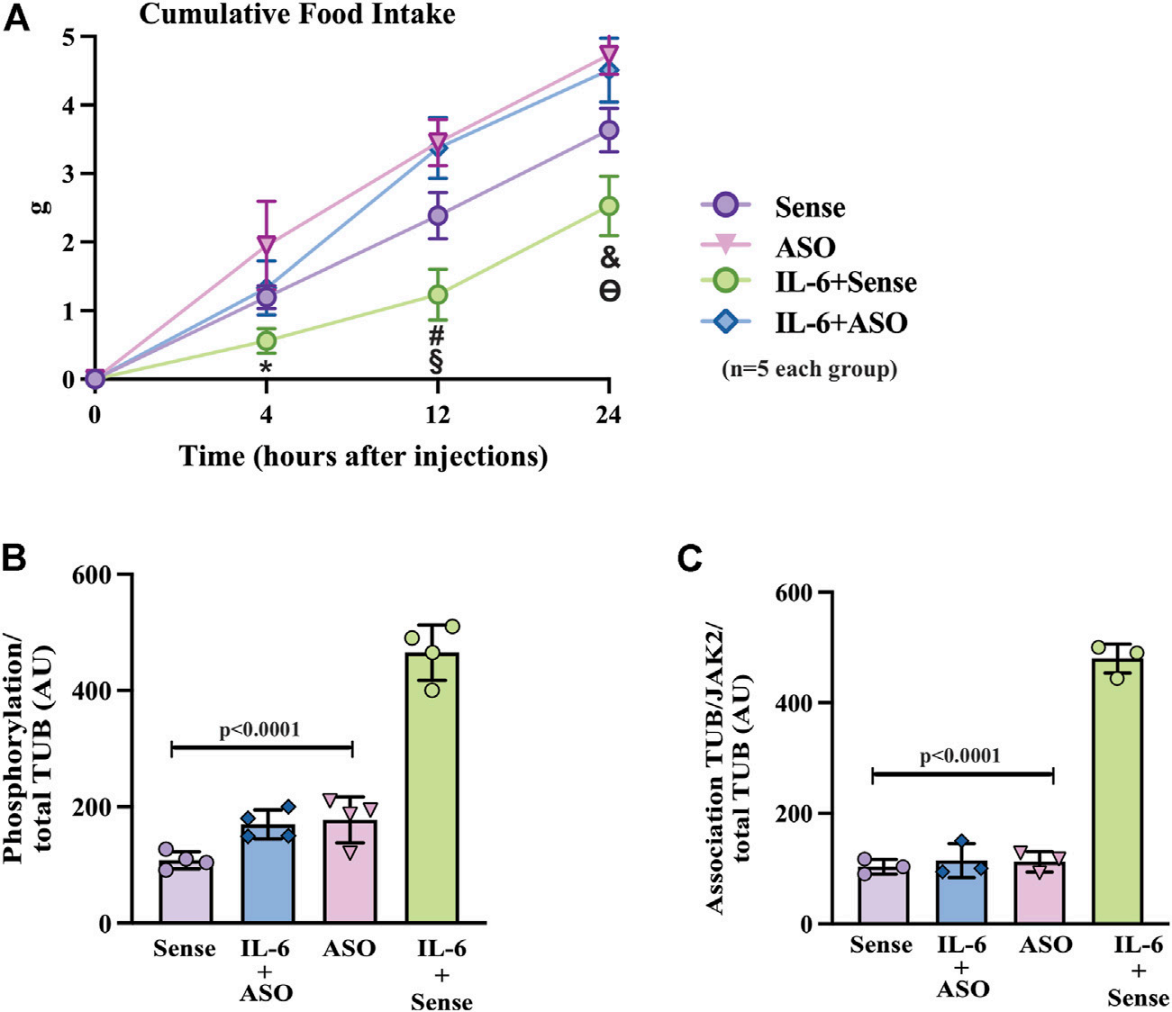
Effects of TUB knockdown on food intake in response to IL-6 ICV injection. **(A)** Evaluation of food intake (g) at 4 h, 12 h, and 24 h in lean mice under resting conditions treated ICV with sense; IL-6+ASO; ASO; IL-6+sense (*n* = 5 each group). Representative quantification of **(B)** TUB tyrosine phosphorylation and **(C)** TUB associated with JAK2 from hypothalamus lysates from lean mice under resting conditions treated with IL-6 ICV or vehicle and pre-treated with ASO against TUB or sense. For this experiment, we used male wt/wt mice (6–8 weeks old) obtained from the B6-tub/tub colony. All values are expressed as means ± standard deviation (SD). Western blot assays (*n* = 3–4 in each group). Two-way ANOVA was used to analyze A, and one-way ANOVA with Tukey’s multiple comparisons tests was used to analyze B and C. * indicates IL-6 vs. Sense, ASO, and ASO plus IL-6 (*p* < 0.0303); **#** indicates IL-6 vs. Sense, ASO, and ASO plus IL-6 (*p* < 0.0042); **§** indicates Sense vs. ASO, and ASO plus IL-6 (*p* <0.0198); **&** indicates IL-6 vs. Sense, ASO,and ASO plus IL-6 (*p* < 0.0092); **ϴ** indicates Sense vs. ASO, and ASO plus IL-6 (*p* < 0.0400).

To further confirm the role of TUB in IL-6 signaling, we knocked down TUB expression in the hypothalamus in another group of sedentary mice using an antisense oligonucleotide (ASO) or sense as control. We then injected IL-6 to measure food intake (Figure 5A). ASO resulted in an 80–90% reduction in TUB expression in the hypothalamus and significantly increased body weight (g) (*n* = 5 in each group) (sense: 16.90 ± 1.57; and ASO: 20.56 ± 1.51; unpaired *t*-test *p* = 0.0035); fat mass from the epididymal (g/100 g body weight, *n* = 5 in each group) (sense: 0.4053 ± 0.2168; ASO: 0.8122 ± 0.2795; unpaired *t*-tests *p* = 0.0234) and food intake (g) (*n* = 5 in each group) (sense: 3.634 ± 0.317 and ASO: 4.732 ± 0.284; unpaired *t*-test, *p* = 0.0004). As expected, IL-6 injection increased TUB tyrosine phosphorylation (Figure 5B) and TUB/JAK2 association in the hypothalamus (Figure 5C), an effect that was abolished by ASO.

## 4 Discussion

The JAK/STAT3 is the canonical pathway by which IL-6 acts in the hypothalamus. Our study is the first to demonstrate an alternative signaling pathway for IL-6 in the hypothalamus through the activation of the tyrosine kinase JAK2, which induces TUB tyrosine phosphorylation. Therefore, TUB is a significant downstream substrate of IL-6. Moreover, our *in vivo* data showed that reduced food intake in response to acute exercise depended on IL-6 signaling through this pathway in the hypothalamus.

IL-6 is classically considered a pro-inflammatory cytokine; it is also the most studied myokine involved in the metabolic benefits of exercise (Pedersen, 2019). IL-6 signaling is complex and crosstalk among pathways can have antagonistic effects depending on the context and cell type expressed (Shirazi et al., 2013; Mauer et al., 2015; Pedersen, 2019; Henning, 2021). In these circumstances, the increased IL-6 levels might be detrimental and correlated with metabolic syndrome and insulin resistance in humans and mice (Mohamed-Ali et al., 1997; Kim et al., 2004; Kim et al., 2012; Wedell-Neergaard et al., 2018). In contrast, during exercise, IL-6 is released by muscle cells and increases up to 100-fold over the basal levels (Pedersen and Febbraio, 2012; Pedersen, 2019). Therefore, the sources of IL-6 secretion play a role in determining the effects of this cytokine in the body. Increased secretion of IL6 from the adipose tissue induces “chronic low-grade inflammation” and insulin resistance (Ropelle et al., 2010; Febbraio, 2017; Pedersen, 2019). Conversely, IL-6 secretion from skeletal muscle during exercise (Pedersen and Febbraio, 2012; Febbraio, 2017; Pedersen, 2019) triggers anti-inflammatory cytokines release and ameliorates glucose metabolism (Pedersen and Febbraio, 2012). In the exercise scenario, despite the presence of obesity, in both mice and humans, increased IL-6 levels potentially enhance insulin and glucagon-like peptide-1 (GLP-1) secretion, improving glucose tolerance and insulin sensitivity (Ellingsgaard et al., 2011; Benrick et al., 2012; Lang Lehrskov et al., 2018; Wedell-Neergaard et al., 2019; Ellingsgaard et al., 2020). The increased levels of IL-6 in obesity may act as a compensatory mechanism to improve insulin resistance instead of inducing chronic low-grade inflammation (Pedersen and Febbraio, 2012). Moreover, our group recently demonstrated the production of IL-6 in the hypothalamus after acute exercise (Katashima et al., 2022). However, which kinds of hypothalamic cells produce IL-6 in response to exercise remains unknown.

In the hypothalamus, IL-6 regulates appetite, energy expenditure, and body composition (Wallenius et al., 2002a; Wallenius et al., 2002b; Mauer et al., 2015; Timper et al., 2017; Katashima et al., 2022). Long-term ICV delivery of recombinant adeno-associated viral vector expressing IL-6 decreased the body weight of male Sprague-Dawley rats through thermogenesis activation without altering food ingestion. In another model, IL-6-deficient mice became obese in adulthood (Wallenius et al., 2002b; Li et al., 2002), partially due to increased food intake (Wallenius et al., 2002b). The specific deletion of IL-6 in skeletal muscle altered the timing of refeeding after overnight fasting (Molinero et al., 2017), suggesting muscle-brain crosstalk. Because exercise increases IL-6 levels up to 100-fold over the basal levels (Pedersen and Febbraio, 2012; Pedersen, 2019), the muscle IL-6 knockout mice refeeding phenotype suggests that high levels of muscle-derived IL-6 play a critical role in suppressing feeding. Timper et al. (Timper et al., 2017) reported a 35% decrease in the food intake of control mice (C57BL/6) exposed to a chow diet after receiving an ICV injection of IL-6. In another study, Silva et al. (Silva et al., 2018) showed that a single ICV injection of IL6 reduced feeding in control mice. In an elegant and recent paper, Katashima et al. (2022) showed that hypothalamic IL-6 activated ERK1/2 (extracellular signal-regulated kinase) signaling in the VMH (ventromedial hypothalamus), which increased fatty acid oxidation *via* AMPK/ACC (5′ AMP-activated protein kinase/acetyl-CoA carboxylase) signaling in the skeletal muscle of mice. Our data demonstrated that IL-6 production did not increase in C3H/HeJ mice in response to acute exercise (Ropelle et al., 2010; Hua et al., 2018); moreover, these mice showed a lower level of decreased food intake compared to that in the control C3H/HePas mice. However, ICV IL-6 resulted in reduced food intake, suggesting that the reduction of feeding after acute exercise depended, at least in part, on IL-6.

The hypothalamic nuclei, including the arcuate hypothalamic nuclei (ARH), VMH, paraventricular hypothalamic nuclei (PVH), and lateral hypothalamus (LH) are enriched in IL-6R (Schéle et al., 2013; Grossberg et al., 2010; Janoschek et al., 2006; le Foll et al., 2015; Benrick et al., 2009). Furthermore, neurons involved in the regulation of energy homeostasis also express IL-6R (Benrick et al., 2009; Grossberg et al., 2010; Schéle et al., 2013). In parallel, IL-6 trans-signaling can occur in neurons that do not express IL-6R (März et al., 1998; Mauer et al., 2015) since the sIL-6R can bind to gp130, which is broadly expressed by cells (Lust et al., 1992; Mauer et al., 2015; Timper et al., 2017). An elegant study demonstrated that the anorexigenic effect of central IL-6 is preserved in obese mice despite leptin resistance (Timper et al., 2017). These results suggest the value of exercise, even in the context of obesity, because the anorexigenic effect of IL-6 is persistent and can help in weight reduction.

Although we observed decreased AgRP and increased POMC mRNA expression in the ARH in the exercise and IL-6-injected groups showed, these findings are not uniform in the literature. Recently, Bunner et al. demonstrated increased feeding, AgRP activation (c-fos) in the ARH, and no effect on the POMC neurons following acute moderate-intensity exercise (Bunner et al., 2020). This discordance might be due to the different exercise protocols. While we applied a swimming protocol (Lenhare et al., 2017; Silva et al., 2018), the other study used treadmill exercise (Bunner et al., 2020). Moreover, the time of day could influence the outcome, as we conducted our exercise and treatments in the light cycle (between 03:00 p.m. and 06:00 p.m.) and evaluated all parameters in the dark cycle (after 06:00 p.m.). However, consistent with our results, another electrophysiology-based study demonstrated that POMC neurons were depolarized and showed enhanced firing while NPY neurons displayed an inhibition phenotype after exercise (He et al., 2018).

The results of the present study reinforced the link between IL-6, exercise, and TUB as the B6-*tub/tub* mice showed a loss of the anorexigenic effect for both IL-6 and exercise. We used B6-*tub/tub* mice 6–8 weeks of age, before obesity was induced, to avoid bias (Coleman and Eicher, 1990; Kleyn et al., 1996). Due to a nucleotide substitution in the 3’ coding region of *TUB* in these mice (Coleman and Eicher, 1990), a splicing disability affects the 44 C-terminal amino acids of the wild-type protein, leading to transcript generation (Noben-Trauth et al., 1996). Thus, some antibodies against the C-terminal may also have detected the transcript in these mice (Stretton et al., 2009; Kim et al., 2017). Although the antibody might detect a signal, Stubdal et al. (Stubdal et al., 2000) developed a *TUB* knockout mouse with an identical phenotype to that of B6-*tub/tub* mice (Stubdal et al., 2000), suggesting that the mutated *TUB* allele did not retain residual function. The authors also observed mutant protein expression in lysates from B6-*tub/tub* mice (Stubdal et al., 2000).

To further strengthen the link between TUB and IL-6 and exercise, we used C3H/HeJ mice with reduced IL-6 production in response to exercise (Ropelle et al., 2010). After exercise, these mice showed lower tyrosine phosphorylation of TUB and TUB/JAK2 association in the hypothalamus compared to the control C3H/HePas mice. IL-6 pre-treatment restored this signal, supporting the crucial role of the IL-6/TUB signaling pathway in regulating feeding after acute exercise.

Another study demonstrated that TUB is a substrate of JAK2 *in vitro* (Kapeller et al., 1999). Our findings support the hypothesis that IL-6 requires JAK2 to increase tyrosine phosphorylation of TUB *in vivo* once AG490 inhibition of JAK2 abolishes TUB phosphorylation in response to IL-6.

Previous studies reported that TUB has a COOH-terminal, a DNA-binding domain, and a regulator NH2-terminal portion, which are compatible with the structure of a transcription factor (Boggon et al., 1999). Moreover, acetylcholine and serotonin induced TUB translocation from the plasma membrane to the cellular nucleus *via* their G-protein receptors (Santagata et al., 2001). The results of these studies suggested that TUB might function as a transcription factor. Therefore, changes in AgRP and POMC mRNA levels in response to exercise might partially be favored by TUB. However, this effect requires additional investigation.

In obese mice with leptin resistance, acute physical exercise such as treadmill or swimming improved leptin signaling in the hypothalamus (Ropelle et al., 2010; Gaspar et al., 2019). Our data suggested that IL-6 may play a role in this process (this effect is probably mediated through IL-6) as the inhibition of IL-6 with AB abolished the anorexigenic effect of leptin plus exercise in diet-induced obese mice (Ropelle et al., 2010). Moreover, the ability of ICV delivery of IL-6 to decrease food intake was preserved in mice on HFD despite leptin resistance (Timper et al., 2017). Together, these data suggest that IL-6 released due to exercise overlaps with the anorexigenic effect of leptin in the context of diet-induced obesity. Likewise, in the present study using mice on a standard diet, either exercise or ICV injection of IL-6 decreased feeding to the same degree as ICV leptin. Exercise, IL-6, and leptin produced similar increases in TUB tyrosine phosphorylation. However, the effects of exercise on the IL-6/TUB pathway in the hypothalamus of obese mice require future investigation. The most important finding of the present study was that ICV leptin after exercise had no additive effect with IL-6 on food intake and TUB regulation in lean mice.

**FIGURE 6:**
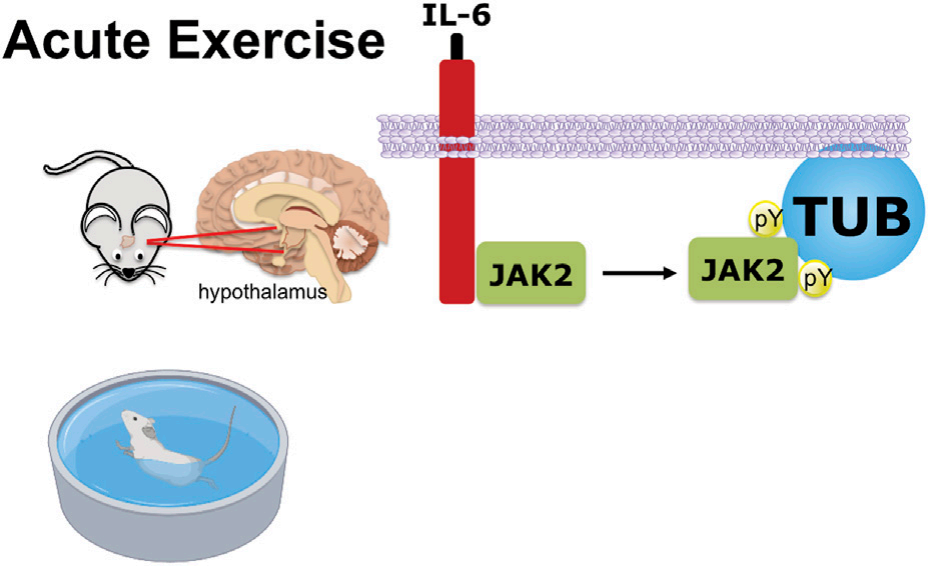
Graphical representation of exercise/IL-6 via the JAK2/TUB axis in the hypothalamus. Acute exercise (swimming) or IL-6 treatment ICV induces TUB tyrosine phosphorylation and its association with JAK2 in the hypothalamus of mice. The activation of the IL-6/Tubby axis reduces food intake. Therefore, acute exercise or IL-6 *via* the activation of the IL-6/JAK2/Tubby axis reduces feeding.

In conclusion (Figure 6), we demonstrated a new pathway reinforcing the benefits of acute exercise, in which the release of IL-6 stimulates JAK2 to increase tyrosine phosphorylation of TUB in the hypothalamus. The anorexigenic effect in response to exercise is, at least in part, linked to this pathway and independent of leptin effects.

## Data Availability

The datasets presented in this study can be found in online repositories. The names of the repository/repositories and accession number(s) can be found in the article/Supplementary Material.
