# Supplementary figures and images for: Acute exercise reduces feeding by activating IL-6/Tubby axis in the mouse hypothalamus
Source: Front Physiol. 2022 Nov 14;13:956116. doi: 10.3389/fphys.2022.956116 (PMC9702993; doi:10.3389/fphys.2022.956116)

Supplemental Figure 8

Figure 4 B

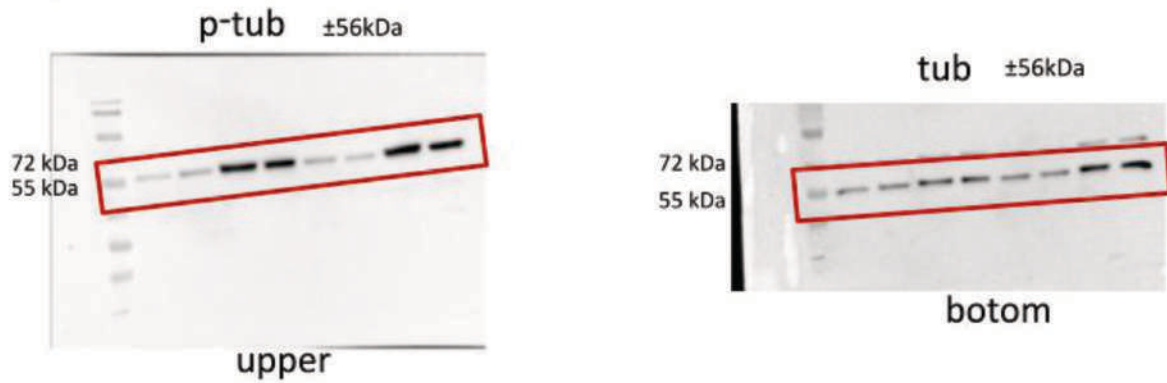

Figure 4 C

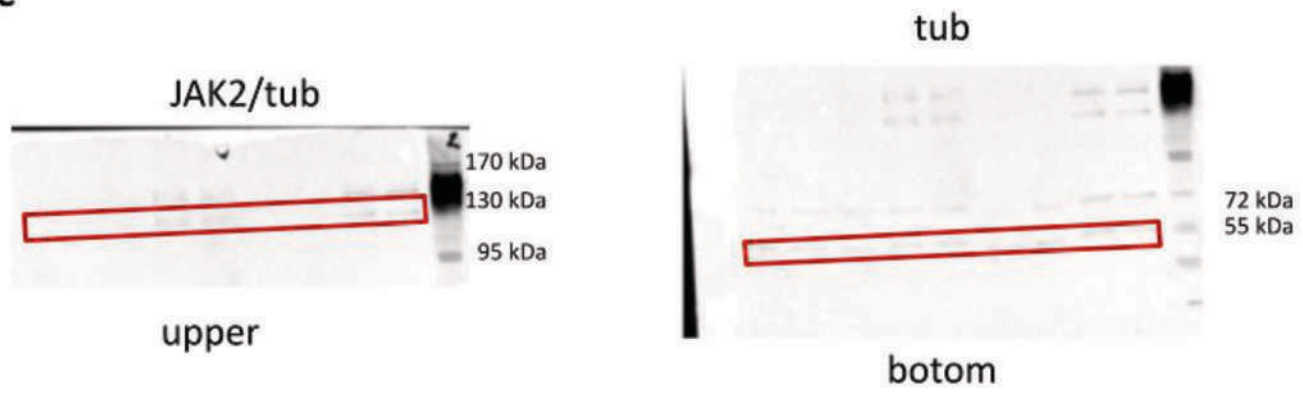

Figure 4E

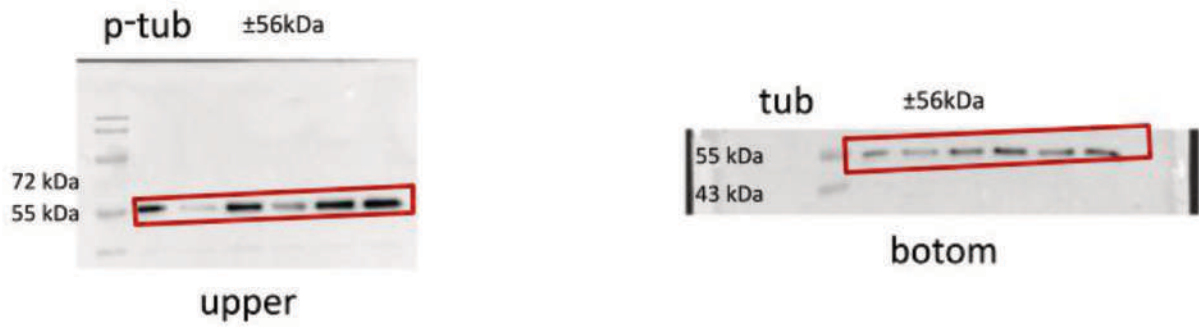

Figure 4 F

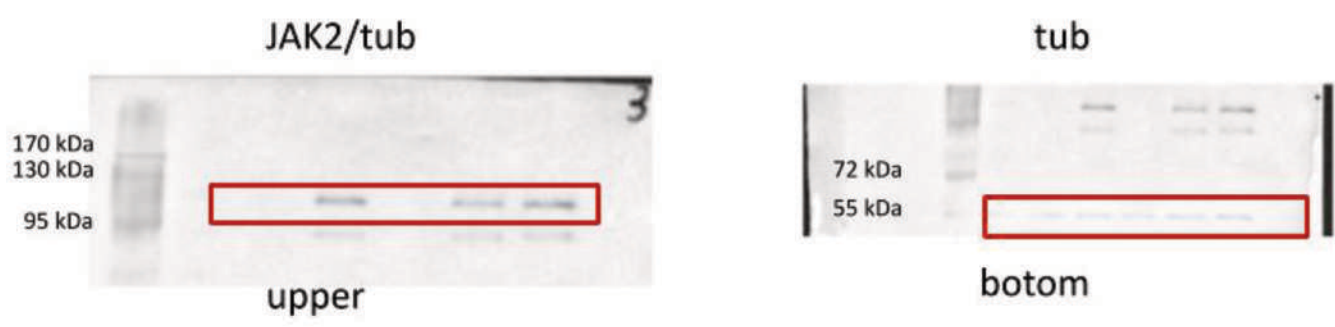

Supplement: Supplementary file 1 [file DataSheet2.PDF]

Supplemental Figure 7

Figure 1

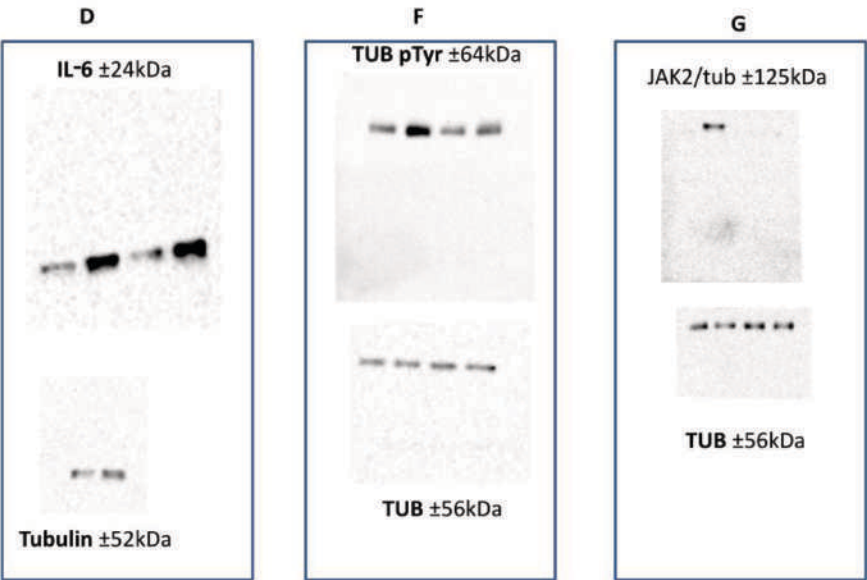

Figure 2

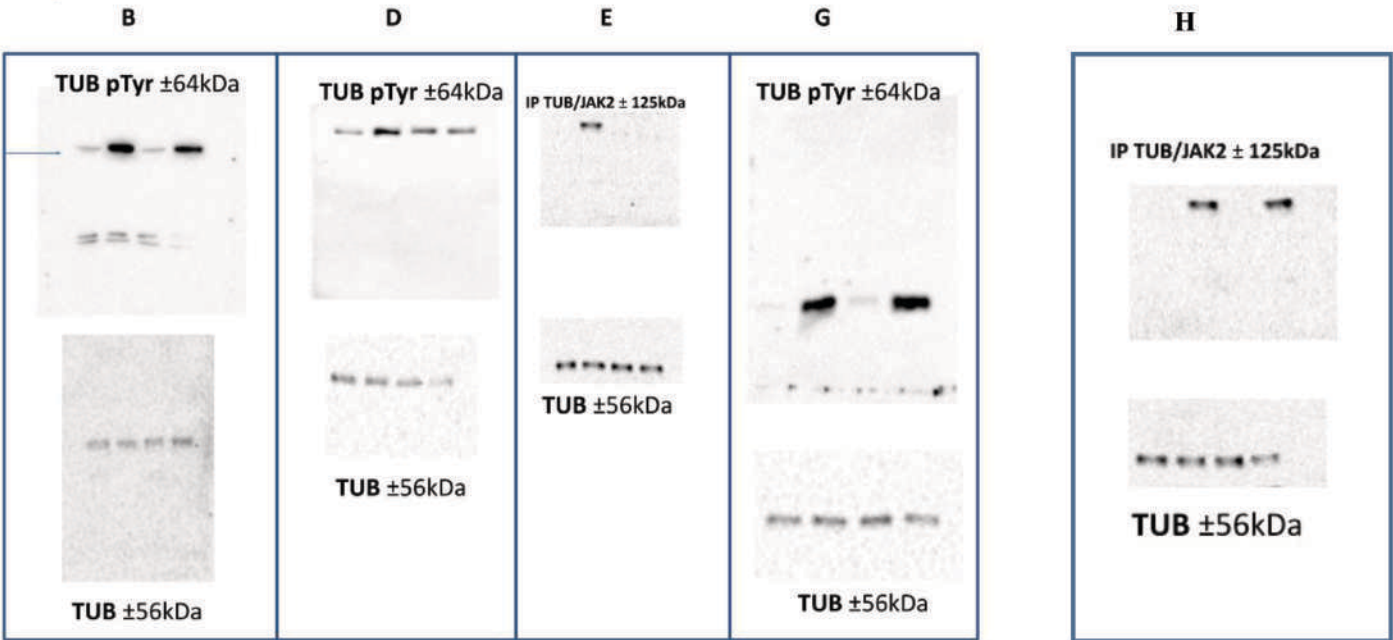

Figure 3

Figure 3 B

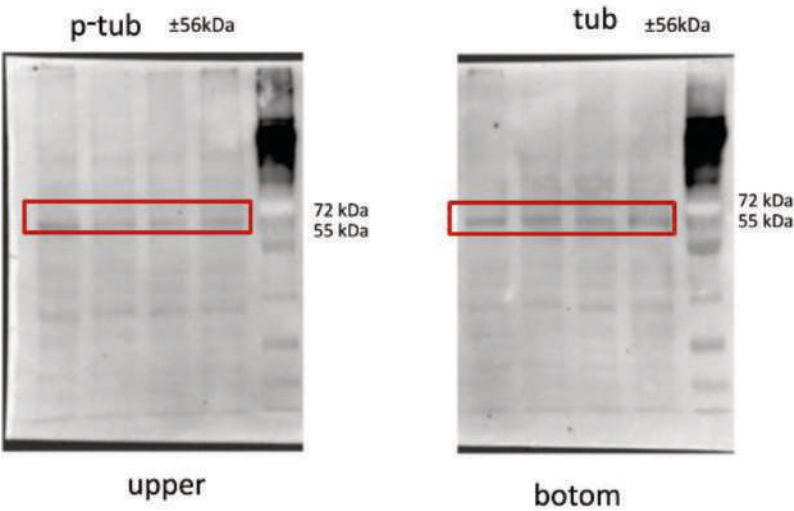

Figure 3 C

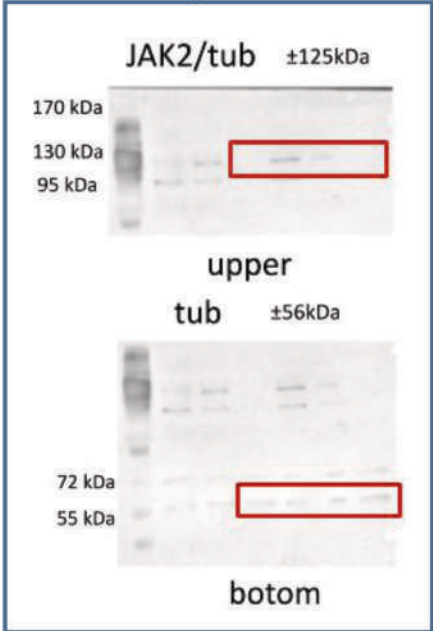

Supplement: Supplementary file 2 [file DataSheet1.PDF]
